# Graphite Compactness Degree and Nodularity of High-Si Ductile Iron Produced via Permanent Mold versus Sand Mold Casting

**DOI:** 10.3390/ma15082712

**Published:** 2022-04-07

**Authors:** Denisa-Elena Anca, Iuliana Stan, Iulian Riposan, Stelian Stan

**Affiliations:** Materials Science and Engineering Faculty, Politehnica University of Bucharest, 060042 Bucharest, Romania; denisa_elena.anca@upb.ro (D.-E.A.); iuliana.stan@upb.ro (I.S.); iulian.riposan@upb.ro (I.R.)

**Keywords:** high-Si ductile iron, solidification, metal mold, sand mold, thin-wall casting, structure, graphite, graphite compactness degree, graphite shape factors, graphite nodularity

## Abstract

In recent years, high-Si ductile cast irons (3–6% Si) have begun to be used more and more in the automotive and maritime industries, but also in wind energy technology and mechanical engineering. Si-alloyed ferrite has high strength, hardness and oxidation and corrosion resistance, but it has low ductility, toughness and thermal conductivity, with graphite as an important influencing factor. In this study, 4.5% Si uninoculated ductile iron solidified in thin wall castings (up to 15 mm section size) via a permanent (metal) mold versus a sand mold, was evaluated. Solidification in a metal mold led to small size, higher graphite particles (less dependent on the section size). The graphite particles’ real perimeter was 3–5% higher than the convex perimeter, while the values of these parameters were 41–43% higher in the sand mold. Increasing the casting section size led to an increased graphite perimeter, with it being much higher for sand mold. The graphite particles’ shape factors, involving the maximum and minimum size, were at a lower level for metal mold solidification, while by involving the difference between P_r_ and P_c_, is higher for the metal mold. The shape factor, including the graphite area and maximum size, had higher values in the metal mold, sustaining a higher compactness degree of graphite particles and a higher nodularity regarding metal mold solidification (75.5% versus 67.4%). The higher was due to the graphite compactness degree level (shape factor increasing from 0.50 up to 0.80), while the lower was due to the graphite nodularity for both the metal mold (39.1% versus 88.5%) and the sand mold (32.3% versus 83.1%). The difference between the metal mold and sand mold as the average graphite nodularity increased favored the metal mold.

## 1. Introduction

Cast iron can be considered unique among metallic materials because no other material has such a long history and such a great diversity of types, properties and applications. Where it is necessary to optimize the specific properties to obtain a piece, cast iron with different graphite shapes in different areas is used. Cast iron is a material that has been developed for thousands of years; while it still has unknowns, it also presents fascinating opportunities [1].

While sharp graphite flakes (the natural graphite morphology in industrial cast iron) create stress concentration points within the metal matrix, rounded nodules inhibit the creation of cracks, thus providing enhanced ductility. The modification of the graphite shape from flakes to nodules has a profound effect on the properties and the market potential of cast iron. In comparison to gray iron, the tensile strength tripled, the stiffness increased by 50% and ductility or elongation changed from close to 0% to more than 5% and up to more than 25% with a ferritic matrix [1,2,3].

Nodular/spheroidal graphite instead of lamellar/flake graphite in cast iron was obtained for the first time in 1943 by Keith Millis, while in October 1949, Keith Dwight Millis, Albert Paul Gagnebin and Norman Boden Pilling obtained the first patent in this field for the production of a cast ferrous alloy for ductile iron using a Mg treatment, according to US patent 2485760, Cast Ferrous Alloy, issued 1949–10–25.

The graphite nucleation sites are forced to grow with a nodular/spheroidal morphology as a result of a metallurgical treatment applied to the iron melt before its solidification, usually consisting of two steps: nodularization/spheroidizing treatment (Mg, Ce, La, Y etc.), followed by a graphitizing treatment/inoculation (Ca, Sr, Ba, etc.).

Ductile iron is greatly used in the automotive industry, where the strength must surpass that of aluminum but steel is not necessarily required. Other major industrial applications include off-highway diesel trucks, agricultural tractors and oil well pumps. In the wind power industry, ductile iron is used for hubs and structural parts, such as machine frames. A large part of the annual production of ductile iron is in the form of ductile iron pipe, which is used for water and sewer lines. Ductile iron is also suitable for large and complex shapes and high (fatigue) loads [4].

Ductile (nodular or spheroidal graphite) cast irons, generally with up to 3.0% Si, are characterized by the highest mechanical properties in the large cast iron family, depending on the metal matrix class. Supplementary silicon alloying (3–6% Si) leads to a ferritic matrix and improved properties, such as strength, oxidation and corrosion resistance. Three Si-alloyed ductile cast iron grades are present in international standard ISO 1083-2018, namely, 3.2% Si (450–18), 3.8% Si (500–14) and 4.3% Si (600–10), as solid-solution strengthened ferritic spheroidal graphite cast irons [5]. This development has led to a significant increase in industrial applications in recent years, such as the automotive industry, maritime industry, wind power technology and mechanical engineering. Higher silicon content (4–6% Si) and Si-Mo (2.5–5.5% Si and 0.2–2.0% Mo) ductile cast irons are usually used for resistance to oxidation and corrosion at high temperatures. More addition leads to superior mechanical properties, especially at high temperatures (Rm = 400–650 MPa, Rp0.2 = 250–550 MPa, A = 3–12%), typically for exhausted applications [6,7].

## 2. Background

A large number of papers reported an inverse silicon segregation pattern, with silicon being highly segregated in austenite, inside of the eutectic, around graphite nodules. In this way, carbon diffusion from austenite to graphite is favored, producing ferrite, starting at the area with the graphite contact (at a high enough silicon content, ferrite will be formed at an increased distance from graphite particles). It was reported that the area around graphite with Si segregation will act as the initiation of and help to propagate crack fractures [8]. This phenomenon was recently shown to be attributed to the formation of iron–silicon long-range orderings that lead to an embrittlement of the material [9]. As a fully ausferritic structure in a Si-solution strengthened matrix, severe Si segregation reduces the stability of C-stabilized austenite and compromises machinability [10]. Silicon segregations in high-silicon alloyed with ductile iron could be specifically manipulated with small additions of aluminum (max. 0.2% Al). Results showed that the fatigue strength can be increased compared to a reference alloy with no aluminum [11].

Silicon is one of the elements that tend toward chunky graphite formation. By comparing the effect of Si and Ni on chunky graphite in heavy castings (micro/macro analysis), it was found that higher Si has a strong influence on chunky graphite formation, especially in castings with a slow cooling rate, while Ni promotes chunky graphite only in the thermal center of the casting (the lowest cooling rate). As result, elongation was severely limited when chunky graphite appeared in the microstructure [12]. Chunky graphite, which is a known defect in high-Si ductile iron, is commonly counteracted using Ce/Sb [13]. In particular, Bi-containing inoculants were shown to be successful in Si alloyed ductile iron [14,15].

Recently [16], an experimental study was undertaken to investigate the effect of silicon and rare earth content on the amount of chunky graphite. It was found that the controlled content of rare earths is beneficial for the reduction in chunky graphite when using standard charge materials.

Tin is known to reduce the appearance of chunky graphite and it was shown that this effect is not related to rare earths. Comparing the effect of tin and antimony, it was observed that although both suppress chunky graphite, they also lead to spiky graphite when rare earths are not added. It was observed that the mechanical properties at room temperature are adversely affected by chunky graphite, more so in the case of low-silicon spheroidal graphite cast iron compared to those with high silicon content. Furthermore, spiky graphite was found to be much more harmful and should therefore be avoided.

Recently [6,17,18,19], it was found that graphite nodularity is negatively affected by high Si content. Especially for more than 4% Si content, a medium-quality graphite phase is obtained, with V-ISO 945 graphite as the prevalent form and inoculation as a beneficial effect, expressed as the improvement in the graphite particles’ shape factors.

For 4.3–4.6% Si content and solidification in a furan resin sand mold (thermal diffusivity 71 W·s^1/2^/m^2^·K), the cooling rate (wall thickness) led to a lower graphite particle area with a lower standard deviation in graphite particle size and a higher graphite particles count characterizes the thin wall solidification (3.5 mm width of wedge casting) compared to a 10 mm wall thickness. A higher cooling rate for the solidification, specifically for a thin wall section (3.5 mm), led to more very small particles, generally with less than a 200 µm^2^ area [19].

According to Borsato, the solidification time has a significant effect on the microstructure and mechanical properties of solution-strengthened ferritic ductile iron. In particular, it was found that with increasing solidification times, the microstructure becomes coarser and the presence of defects increases. The lower the cooling rate, the lower are the measured tensile and fatigue properties [20].

Different microstructures of EN-GJS-SiMo45-6 were attained for thin-walled castings with wall thicknesses of 3 and 5 mm, as well as reference castings with typical wall thicknesses of 13 and 25 mm, which led to various cooling rates that were calculated [21]. The study showed that the cooling rates of castings varied within a wide range (27 °C/s–1.5 °C/s) when considering wall thicknesses of 3 to 25 mm. Increasing the cooling rate reduces the C and Mo segregation to the intercellular regions and, hence, reduces the number of intercellular precipitates. These results suggest that the occurrence of pearlite and carbides is related to segregations during solidification rather than to cooling rates at the eutectoid temperature.

According to Alonso, a higher nodule count, associated with a higher cooling rate at the end of solidification, generates lower porosity. To determine the nuclei’s nature, SEM analysis was undertaken, and as the main graphitizing germs, complex nitrides of (MgSiAl)N were found [22].

The as-cast microstructure of 3.2–4.3% Si ductile iron solidified in 300–450 °C preheated gray iron mold (10–30 mm wall thickness castings) was analyzed [23]. The results show that an as-cast microstructure free from carbides is achieved in sections of 20 and 30 mm, whereas for 10 mm, a partially chilled microstructure is produced. As expected, the pearlite content decreases with increasing silicon content and increasing preheating temperature. A higher cooling rate increases eutectoid undercooling and thus promotes pearlite formation. From the experiments carried out, a quantitative correlation was deduced for the pearlite content as a function of eutectoid undercooling and Si content. The nodule count was approximated as a linear dependency on solidification time.

A previous paper [24] evaluated the solidification pattern of 4.5% Si uninoculated ductile cast iron via standard cooling curve analysis and addressed the effects of the solidification cooling rate on an as-cast structure as determined by metal mold versus green sand mold casting and section size (3–15 mm). Metal mold solidification produces an increase in graphite nodularity and nodule count, lowering the graphite and ferrite amount, with a presence of up to 15% carbides compared to a sand mold.

It appears that high-Si ductile iron solidified in a metal mold is more sensitive to pearlite and carbide stabilizing elements [24] than in a sand mold, documented as a capacity to tolerate them [14,15,25,26,27]. Considering that steel scrap containing carbide-promoting elements (Mn, Cr, Mo, Nb, V) is the most important feedstock for high-Si ductile iron production, recent work [28] studied the impact of these elements on the mechanical properties of ductile iron EN-GJS-500-14 with 3.8 wt.% silicon. The results showed that Cr and V led to the highest strengthening in this ductile iron in relation to their alloy content. The increasing amount of pearlite and carbides in the microstructure produced a tensile strength increase, while the increasing solidification time produced a decrease in all mechanical properties.

As it was found that at a higher silicon content (especially at more than 4% Si), a lower compactness degree of graphite particles is produced, and a higher solidification cooling rate, as expressed by wall thickness or/and mold media thermal properties, could be an important influencing factor, the main objective of the present study was to evaluate the graphite phase characteristics (focused on the graphite particles compactness degree and its influence on the graphite nodularity) of 4.5% Si uninoculated ductile iron, solidified in thin wall castings (up to 20 mm section size), comparing a permanent mold with a green sand mold.

## 3. Materials and Methods

The iron melt was obtained via electric melting (coreless induction furnace, 10 kg capacity, 8000 Hz) of the usual charge, including pig iron, steel and cast iron scrap (FeSi). A nodularization treatment (1525 °C) was recorded using the Tundish cover technique (1.5 wt.% FeSiCa1Mg6RE0.3 alloy), followed by wedge test samples (20–22 mm base, 45–57 mm height) cast in a green sand mold and metal mold (1450 °C) without inoculation. The two types of molds are differentiated via their thermal diffusivity: 14,000 W·s^1/2^/m^2^·K for the metal mold and 1396 W·s^1/2^/m^2^·K for the green sand mold.

A SPECTROLAB high-end spectrometer (Sylmar, CA, USA) with hybrid optic (PMT and CCD were used for a high-precision metal chemical composition analysis. The instrument achieves detection limits below 1 mg/kg. Table 1 includes the final chemical composition of the obtained ductile cast iron, as the base elements (C, Si, Mn, P, S), nodulizing elements (Mg, Ce, La, Ca) and minor elements.

−CE = % C + 0.3·(%Si + %P) − 0.03·(%Mn) + 0.4·(%S)(1)

The graphite characteristics were evaluated with Automatic Image Analysis (OMNIMET ENTERPRISE (Lake Bluff, IL, USA) and analySIS^®^ FIVE Digital Imaging Solutions software) for particles greater than 5 µm in size in an analyzed field area of 0.59 mm^2^.

## 4. Results

### 4.1. Structure Characteristics

**Figure 1:**
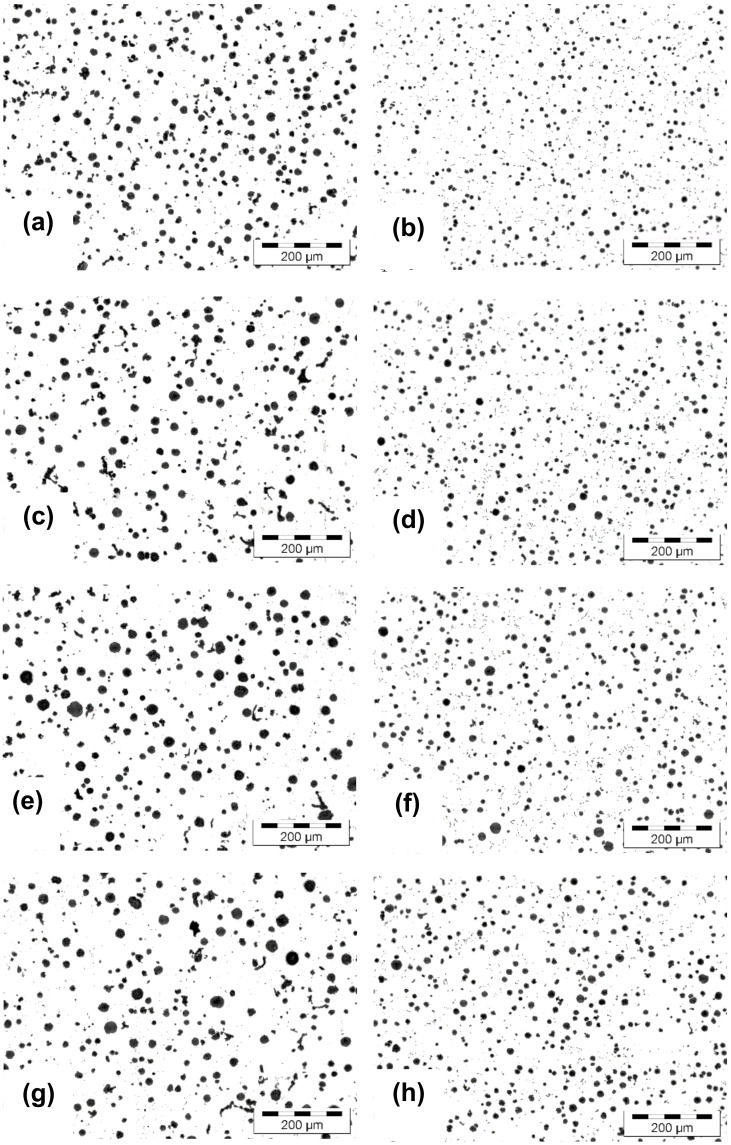
Structure of 4.5% Si ductile iron solidified in wedge casting in a green sand mold (**a**,**c**,**e**,**g**) and metal mold (**b**,**d**,**f**,**h**) with different wall thicknesses: ((**a**,**b**) 6 mm; (**c**,**d**) 9 mm; (**e**,**f**) 12 mm; (**g**,**h**) 15 mm) [un-etched].

Figure 1 shows the graphite phase aspect as influenced by the mold media (sand mold versus metal mold) and casting wall thickness (6–15 mm section size of wedge casting).

In a previous paper [24], the solidification pattern of this 4.5% Si uninoculated ductile iron was evaluated using standard ceramic cup thermal (cooling curve) analysis that addressed the effects of the solidification cooling rate on structure as determined by changing the thermal parameters of the molding media (metal versus green sand mold) and section size (wedge castings, 1–20 mm wall thickness). It was found that 4.5% Si alloying led to a large ΔTs eutectic interval (closer to 80 °C) and a very low undercooling relative to the stable eutectic temperature (only 11% from ΔT_s_).

The start and the end of the eutectic reaction were away from the metastable (carbidic) eutectic temperature T_mst_ (ca. 70 °C) at 0.5 °C recalescence, while the temperature of the end of solidification was more than 18 °C above this temperature, despite the cast iron not being inoculated.

**Figure 2:**
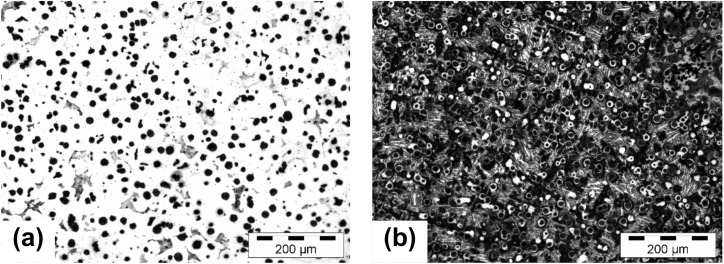
Structure of 4.5% Si ductile iron solidified in wedge casting in a sand mold (**a**) and a metal mold (**b**) with a 6 mm wall thickness [etched].

The graphite amount was up to 65% lower in the metal mold and more depending on the section size. The metal mold versus green sand mold solidification increased the nodule count (690 vs. 523 nodules/mm^2^ on average), with a reduced effect of the wall thickness increase (10 vs. 30% decreasing), and with a 5–10% higher graphite nodularity (88–91 vs. 78–86%). There were no free carbides in the green sand mold but up to 15% carbides in the 6 mm thickness metal mold, with a decrease of up to 5% for the 12–15 mm thickness (Figure 2). In the experimental conditions, 70–100% ferrite was found in the green sand mold and 10–20% ferrite in the metal mold. It was concluded that solidification in the permanent (metal) mold of high-Si ductile cast iron was more sensitive to chemical composition, including minor (trace) elements, than in the sand mold, which was documented as having a high capacity to tolerate higher amounts of pearlite and carbide-stabilizing elements.

**Figure 3:**
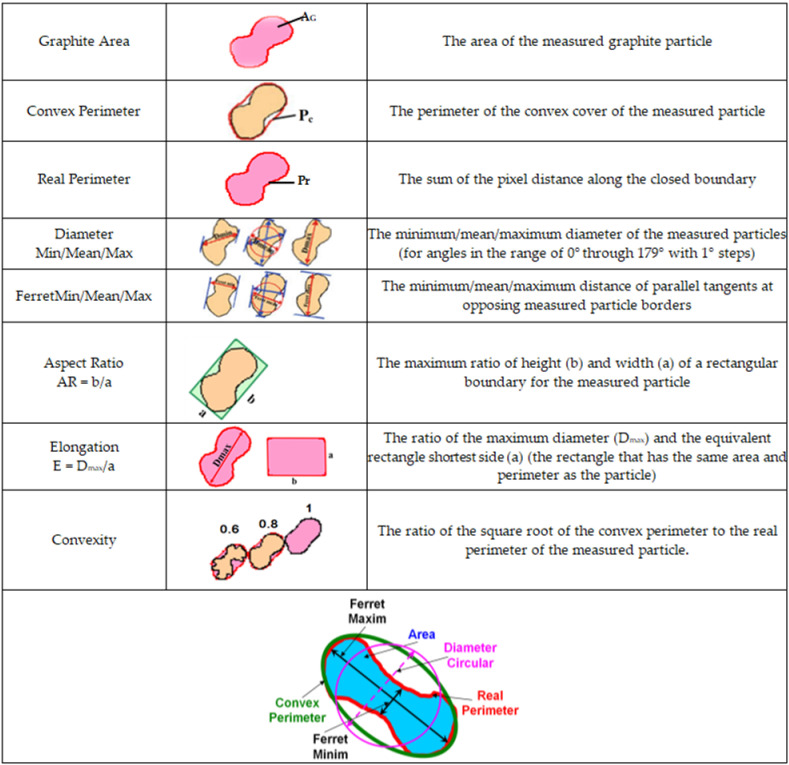
Graphite particle parameters that were evaluated using image analysis.

The present paper reports detailed analysis results of the graphite phase characteristics, mainly as graphite shape factors (including representative graphite particles parameters) and their involvement in the graphite nodularity evaluation (Figure 3).

### 4.2. Graphite Size and Count Characteristics

**Figure 4:**
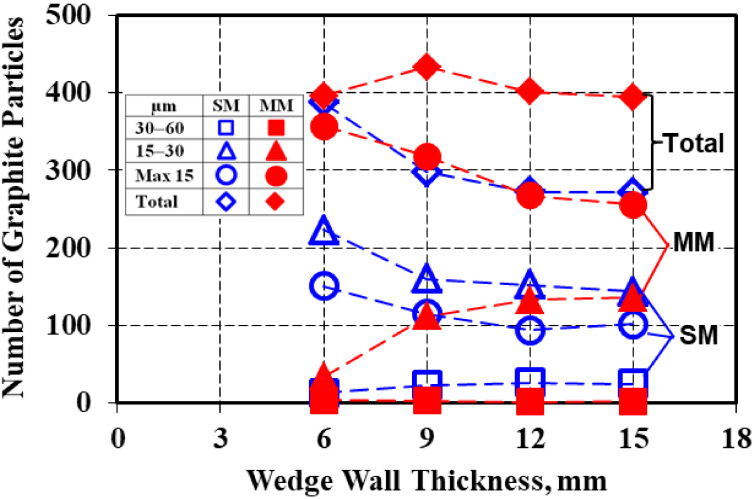
Influence of the mold media type and wedge casting wall thickness on the graphite particles size (max. 15 µm, 15–30 µm, 30–60 µm) distribution [0.59 mm^2^ analyzed field].

According to the Figure 1 microstructures, there was a big difference in graphite phase characteristics, mainly as an effect of the mold media thermal properties but also the wedge casting wall thickness variation (Figure 4). Generally, a higher solidification cooling rate in metal mold led to a higher graphite particles count compared to the sand mold, with the difference increasing as the casting wall thickness increased.

Regarding the nodule count, the metal mold casting was less dependent on the increase of the section size (from 6 up to 15 mm), with 10% fewer nodules compared with 30% fewer nodules in the sand mold in the 15 mm wall thickness. An important difference was found regarding nodule size and its distribution on the casting size. Metal mold casting was characterized by the finest graphite nodules, mainly less than 15 µm size (the rest were 15–30 µm in size).

In contrast, in the sand mold solidification, the 15–30 µm size nodules prevailed, with 30–40% more than the max. 15 µm size nodules and with the presence of 3–10% more larger nodules (30–60 µm). The number of nodules less than 15 µm in size in the metal mold solidification was at the same level as the total number of graphite particles in the sand mold casting.

As previous results [6,17,18,19] pointed out the visible difference between the convex and real perimeters of graphite particles (defined in Figure 3) in high-Si ductile cast iron, a detailed analysis was undertaken for these parameters as a function of the solidification cooling rate, which was determined by the mold thermal properties and casting wall thickness (Table 2).

**Figure 5:**
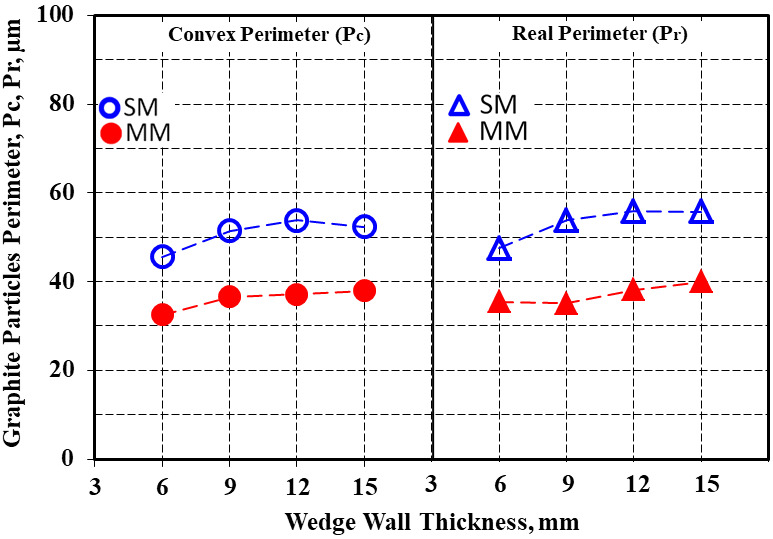
Influence of the mold media and wall thickness on the values of the convex and real perimeters of the graphite particles.

**Figure 6:**
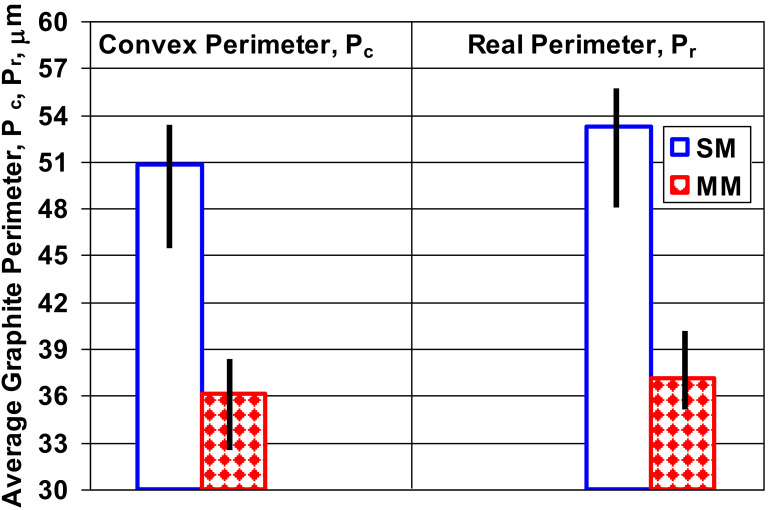
Average convex and real perimeter values of the graphite particles in the 6–15 mm wedge casting section size solidified in the sand and metal molds.

The real perimeter values were higher than for the convex perimeter, with 41% for the sand mold and 43% for the metal mold solidification conditions as average values. The increase in the casting section size, which meant the decrease in the solidification cooling rate from this point of view, favored the increase of the graphite particles’ perimeters (Pc and Pr) much more for the sand mold casting (17–18%) compared to 14–16% in the metal mold (Figure 5 and Figure 6).

**Figure 7:**
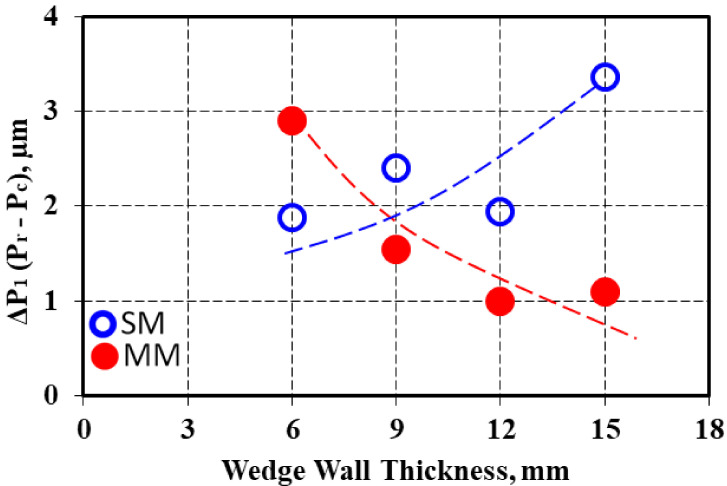
Influence of the wedge casting wall thickness on the difference between the real and convex perimeter (ΔP_1_) for the sand and metal molds.

**Figure 8:**
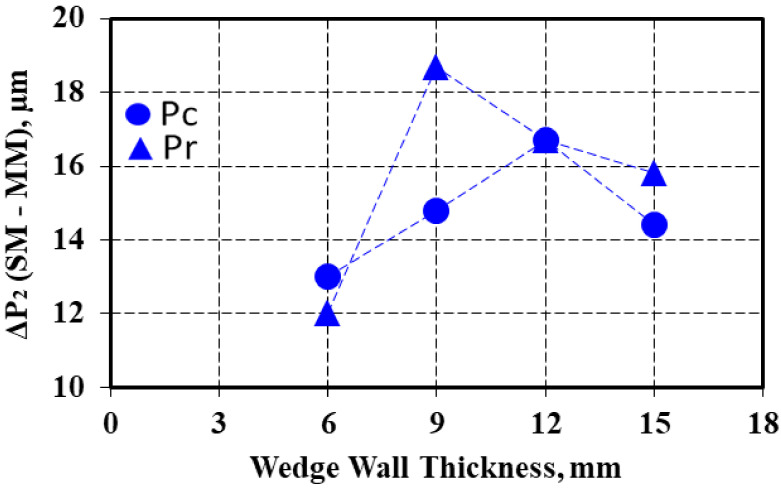
Influence of the wedge casting wall thickness on the difference between the graphite perimeter values (ΔP_2_) in the sand and metal mold solidification.

The difference between the real perimeter and convex perimeter (ΔP1 = Pr–Pc) for the same casting section size increased with wall thickness increases for the sand mold solidification, but this parameter decreased for the metal mold casting (Figure 7). The ΔP2 factor, expressing the difference between the graphite particles perimeter in the sand mold and metal mold castings for the same casting section size was higher for the real perimeter than for the convex perimeter, with the same evolution with the casting wall thickness variation for both the Pc and Pr parameters (Figure 8), with a maximum level found at a 9 mm casting section size for Pr and a 12 mm casting section size for the Pc parameter.

**Figure 9:**
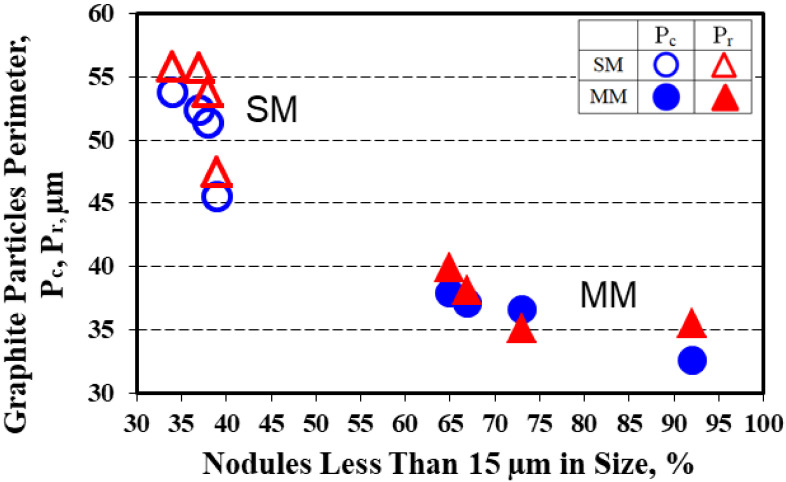
Influence of the nodule size distribution on the graphite particles’ perimeters.

It appeared that the graphite particle perimeter characteristics were related to the graphite phase size and size distribution and were dependent on the mold media and casting section size (Figure 4). The structure of the metal mold casting contained not only a higher level for the nodule count but at the highest incidence of nodules that were less than 15 µm in size (65–90%, the rest were 15–30 µm in size), lower perimeter values and higher compactness degrees were typically found, with a lower difference between the real and convex perimeters (Figure 9).

The higher rate of nodules 15–30 µm in size (32–58%) and the increase in the incidence of larger nodules (5–10%, more than 30 µm) in the sand mold casting (only 34–39% less than 15 µm size) led not only to the increase of both the Pc and Pr parameters but also to the increase in the difference between them, especially at a larger casting section size (lower solidification cooling rate).

### 4.3. Graphite Particles Shape Factors

The graphite particles’ compactness degree, usually expressed as different shape factors, is important for describing the action of the graphite particles as the most important stress concentrator in the cast-iron structure. The higher the graphite compactness degree, the lower the capacity of graphite particles to resist stress concentration and, consequently, all of the mechanical properties will be higher. As the highest compactness degree of an object is characteristic of a sphere, leading to the lowest capacity for stress concentration, the deviation using a sphere as a reference of graphite particles has a negative effect, as it determines the excessive concentration of stress at the graphite–matrix interface.

Table 3 includes the average values of the representative graphite particles’ shape factors, defined in Figure 3, as the effect of the mold material and casting section size.

The first group of shape factors considers the size of graphite particles, expressed using AR and E. A higher compactness degree of graphite particles is expressed by lower values of AR and E shape factors. The roundness shape factor involves two size parameters of the graphite particles, namely, area and the maximum size, (F_max_) and is defined using Equation (2):

RSF = 4·A_G_/π·F_max_^2^ (2)

**Figure 10:**
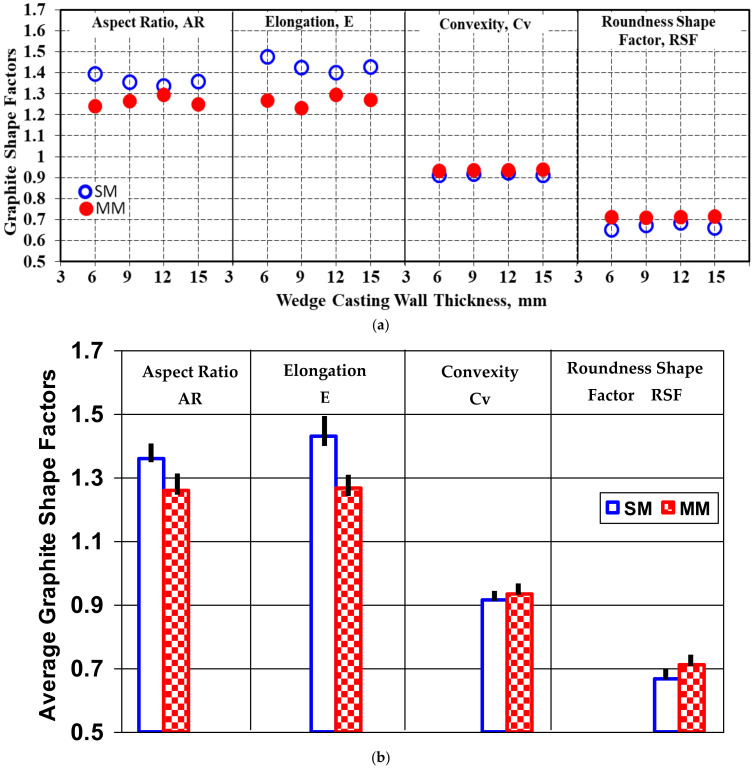
Influence of the mold media and wedge casting wall thickness on the level of the aspect ratio, elongation, convexity and roundness shape factor [(**a**) at different wall thicknesses; (**b**) average for the wedge casting].

Figure 10 shows the level of these graphite shape factors, which was at a lower level for the metal mold solidification (AR = E = 1.2–1.3) compared to the sand mold casting (AR = 1.3–1.4 and E = 1.4 = 1.5) and had a lower dependence on the casting wall thickness in the present test conditions.

The convexity shape factor involves the difference between the real and convex perimeter of graphite particles and refers to the ratio of the square root of the convex perimeter to the real perimeter of the measured particle. This factor has higher values for a lower difference between real and convex perimeter, reaching Cv = 1.0 when P_r_ = P_c_ (see Figure 3). According to Figure 10, Cv = 0.93–0.94 for the metal mold and Cv = 0.91–0.92 for the sand mold casting, with a low effect due to wall thickness variation and a good relationship with obtained data for the convex and real perimeter parameters (Figure 5, Figure 6, Figure 7, Figure 8 and Figure 9).

The roundness shape factor (Equation (2)) is usually used to identify different graphite morphologies in cast iron, from lamellar through vermicular up to nodular graphite, including different sub-classes for each graphite type.

**Figure 11:**
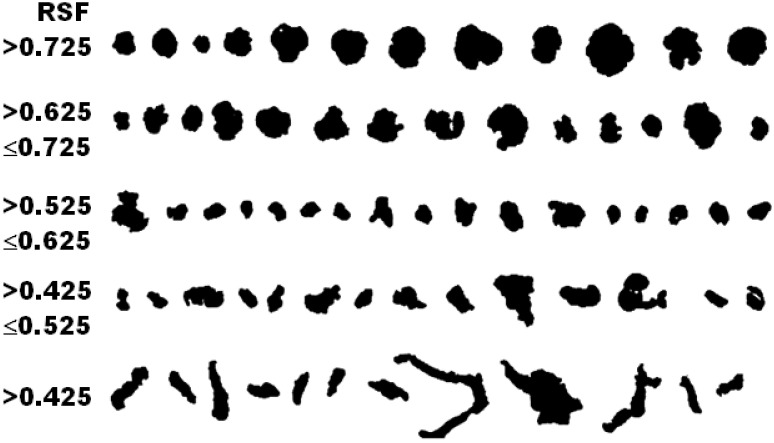
Visual representation of graphite particles categorized using RSF, according to ISO 16112:2017.

International standard ISO 16112:2017 refers to “Compacted (vermicular) graphite cast irons–Classification” [29] and defines some graphite morphologies that could be present in this type of cast iron (Figure 11). In this standard, via the use of the RSF, nodular graphite (ISO form VI) was defined using RSF = 0.625–1.0, with intermediate forms of graphite (ISO forms IV and V) having RSF = 0.525–0.625 and compacted graphite (ISO form III) having RSF < 0.525.

According to the ISO 945-4-2019 international standard, nodular graphite means graphite particles classified as form VI and V in accordance with ISO 945-1 or graphite particles with RSF ≥ 0.6 to 1.0: the 0.6–0.8 range is specific for slightly irregular spheroidal graphite morphology (type V, ISO 945), while RSF > 0.80 refers to higher compactness degree type VI ISO 945 graphite particles [30,31].

The roundness shape factor was at the 0.65–0.75 level in the sand mold solidification and at the 0.71–0.72 level in the metal mold solidified castings, with a small dependence on the casting wall thickness and solidification cooling rate, which were less accentuated for the metal mold.

Generally, the solidification of the 4.5% Si uninoculated ductile cast iron in the metal mold was characterized by better graphite shape factors at lower values for the aspect ratio and elongation and a higher level for the convexity and roundness shape factor.

### 4.4. Graphite Nodularity

Graphite nodularity is an important quality factor of the graphite phase in cast iron that is subjected to a graphite nodularization treatment (Mg, rare earth) before solidification in order to obtain compacted (vermicular) or nodular (spheroidal) graphite cast irons. As a general rule, graphite nodularity expresses the rate of defined nodular (spheroidal) graphite particles in the total present graphite particles in the cast-iron structure. As an example, 80% nodularity could characterize a cast iron with 80% nodular graphite and 20% other graphite morphologies, mainly compacted/vermicular graphite.

As imposed graphite nodularity is one of the most important quality factors and a condition to accept/reject the casting, it is very important to define the term nodular/spheroidal graphite from the point of view of the compactness degree of graphite particles. This is recorded using different graphite shape factors.

**Figure 12:**
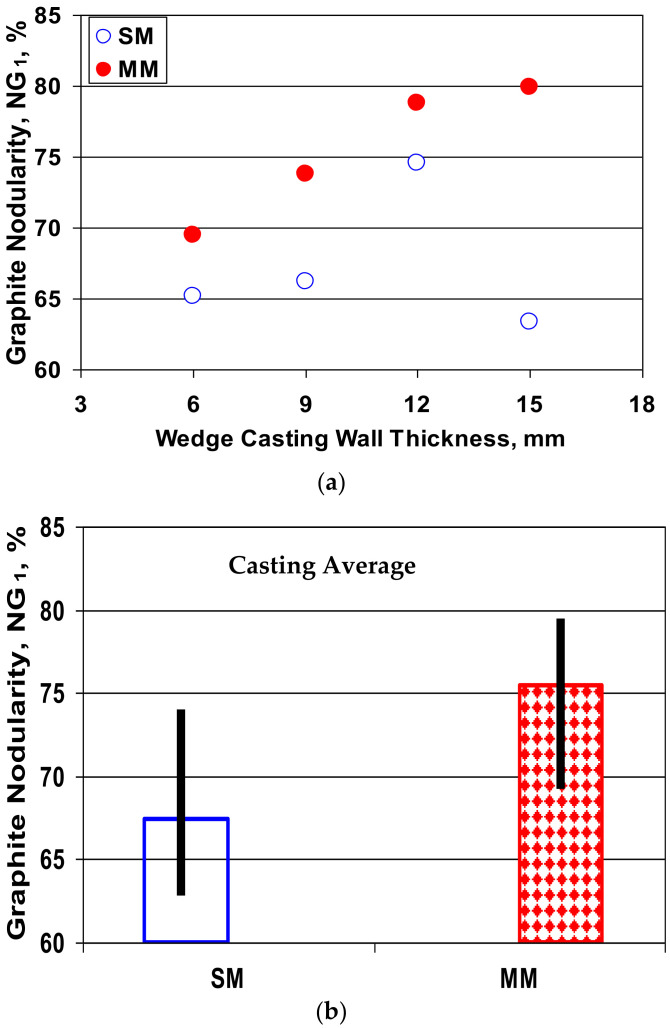
Influence of the mold type and the wedge casting section size on the graphite nodularity NG_1_ (**a**) and the average values on the 6–15 mm section size (**b**).

In the present work, graphite nodularity was calculated according to Equation (3) by considering the roundness shape factor, which involves the maximum graphite particle size (F_max_), Equation (2) and the nodular/spheroidal definition according to the international standard ISO 945-4-2019: RSF ≥ 0.6 to 1.0. The total area of graphite particles with RSF ≥ 0.8 and 90% of the total area of graphite particles with RSF = 0.6–0.8 were considered. Figure 12 shows the obtained results.

(3) $${\mathrm{NG}}_{1}=\frac{\Sigma A_{particles\;\left(\mathrm{RSF}\geq 0.8\right)}+0.9\cdot \Sigma A_{particles\;\left(\mathrm{RSF}=0.6-0.8\right)}}{\Sigma A_{tot}}\times 100\left[\%\right]$$

For the entire section of the wedge casting (6–15 mm section size), NG_1_ = 65–75% for the sand mold solidification and NG_1_ = 70–80% for the metal mold casting. This meant that the graphite phase had a lower compactness degree, generally at the lowest limit of acceptance as ductile cast iron, or nodular graphite cast iron, respectively.

In the test experimental conditions, as the uninoculated and more than 4% Si ductile cast iron solidified with a lower casting section size (up to 15 mm), with graphite nodularity calculated based on the graphite shape factor involving the graphite particles’ maximum size, the graphite nodularity was higher in the metal mold compared with the sand mold and increased with casting wall thickness increasing.

**Figure 13:**
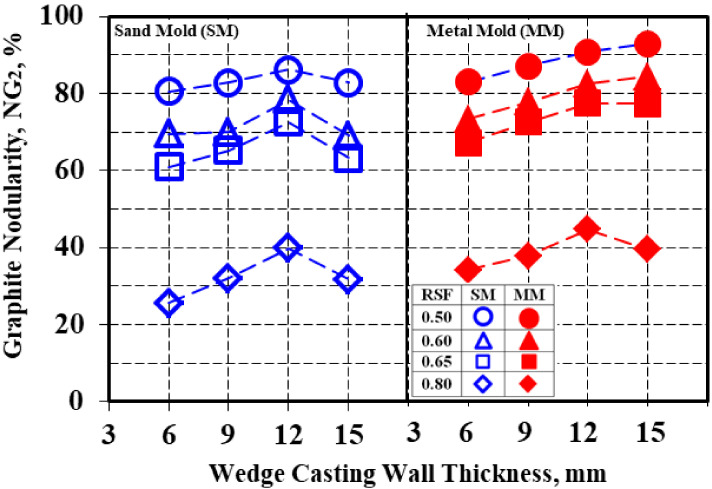
Graphite nodularity (NG_2_) as a function of the mold media and wedge casting wall thickness for imposed minimum values for RSF.

**Figure 14:**
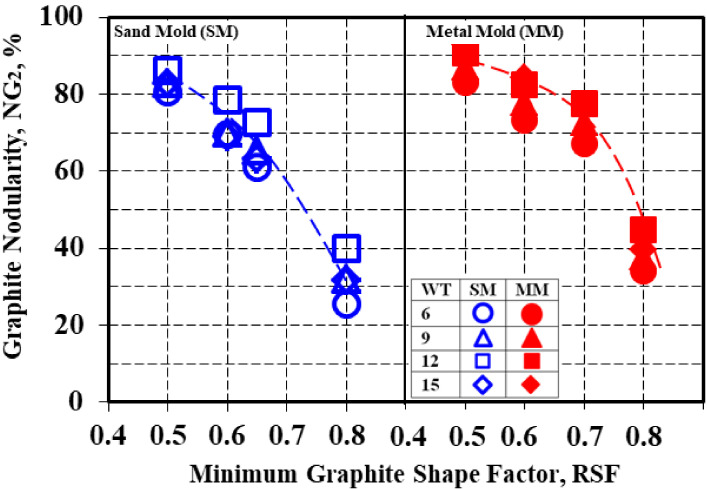
Influence of the minimum imposed RSF on the graphite nodularity NG_2_ as effects of the mold media and wedge casting wall thickness (mm).

**Figure 15:**
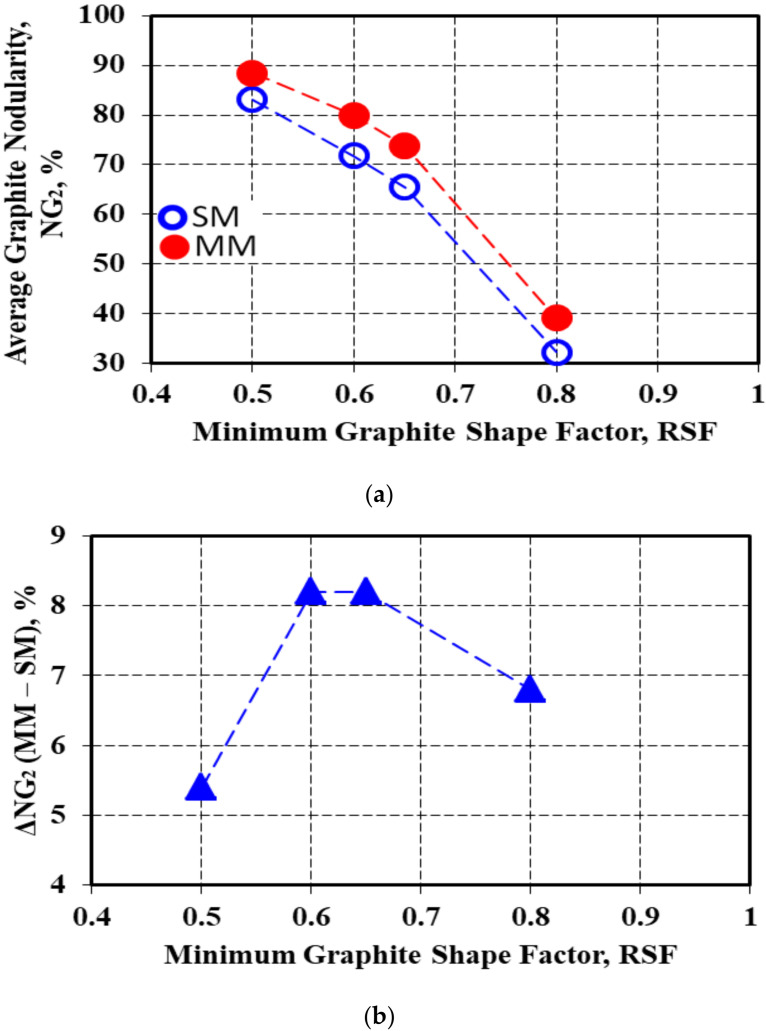
The average graphite nodularity NG_2_ as a function of the imposed minimum graphite shape factor for the sand mold and metal mold solidification (**a**) and the difference between the metal mold and sand mold values (**b**).

The graphite nodularity was strongly dependent on the minimum imposed value for the considered graphite shape factor (RSF), expressed in the present work using NG_2_ (Equation (4)) (Table 4, Figure 13, Figure 14 and Figure 15). The RSFs at the minimum levels of 0.50 (the lowest graphite phase compactness degree), 0.60, 0.65 and 0.80 (the highest graphite phase compactness degree, typically form VI, ISO 945) were considered.

(4) $${\mathrm{NG}}_{2}=\frac{\Sigma A_{particles\left[RSF\right]}}{\Sigma A_{tot}}\times 100\;[\%]$$

Three groups of variation lines could be identified in Figure 13 according to RSF = 0.50, RSF = 0.60–0.65 and RSF = 0.80. Higher is imposed the minimum roundness shape factor RSF, and, consequently, the highest is the graphite particles compactness degree, the lower is resulted calculated graphite nodularity (NG_2_), for both sand mold and metal mold solidification conditions and wedge casting wall thickness, respectively (Figure 14).

Considering the total graphite particles at minimum RSF = 0.50 led to 80–86% nodularity in the sand mold and 83–93% in the metal mold. The nodularity level decreased for RSF = 0.60–0.65, up to 61–78% in the sand mold and 67–85% in the metal mold. The lowest level of nodularity was found for RSF = min. 0.80, with 25–40% for the sand mold and 34–45% for the metal mold.

An increase in the wedge casting wall thickness generally led to an increase in the graphite nodularity level for both the sand mold and metal mold solidification and for all of the imposed conditions regarding the minimum considered roundness shape factor. In the sand mold casting, the maximum graphite nodularity was reached at a 12 mm wall thickness for the entire range of the RSF variation (0.50–0.80).

In the metal mold solidification, a continuous increase in the graphite nodularity was registered for RSF = 0.50–0.60, with the maximum level at a 15 mm casting size. At RSF > 0.60, the maximum level of nodularity was obtained for the 12 mm casting size, similar to the sand mold solidification. The higher the imposed RSF level, the higher the decreasing rate of the graphite nodularity at more than a 12 mm wall thickness.

Figure 15 shows the decrease in the average graphite nodularity NG_2_ of the entire wedge casting as the minimum imposed graphite shape factor (RSF) increased, where the NG_2_–RSF relationship also depended on the solidification conditions (metal mold versus sand mold).

At the lowest imposed graphite shape factor values (SSF = 0.50), the average graphite nodularity was in the 80–90% range, and at a higher level for the metal mold. An increase in the imposed graphite shape factors values led to a decrease in the average graphite nodularity level for all the test conditions: 70–80% for RSF = min. 0.60, up to 65–75% for RSF = min. 0.65 and, finally, 30–40% for RSF = min. 0.80. The results obtained in the metal mold casting remained above the results obtained in the sand mold, but with more scattered values.

The difference between the metal mold and sand mold solidification remained positive for the entire RSF = 0.50–0.80 range, but at the lowest level for the lowest considered graphite compactness degree (RSF = 0.50). The maximum positive effect of the metal mold solidification compared to the sand mold was registered for the intermediary shape factor (RSF = 0.60–0.65).

## 5. Conclusions

The present study evaluated the graphite phase characteristics (focused on the graphite particles’ compactness degree and its influence on the graphite nodularity) of 4.5% Si uninoculated ductile iron that was solidified in thin wall castings (up to 20 mm section size) via a permanent mold versus a green sand mold. The following conclusions were drawn:

(1) A higher solidification cooling rate in the metal mold led to a higher graphite particle count compared to the sand mold, with the difference increasing as the wall thickness increased, but the metal mold casting was less dependent on the increase in the section size.
(2) The metal mold casting was characterized by the finest graphite nodules, mainly less than 15 µm in size (the rest of 15–30 µm size), which were at the same level as the total number of graphite particles in the sand mold casting (with a prevalent 15–30 µm size).
(3) The graphite particles’ real perimeter was 3–5% higher than the convex perimeter, while the values of these parameters were 41–43% higher in the sand mold. The increase in the casting section size favored the increase in graphite perimeter, with it being much more for the sand mold. The difference between the graphite particles’ perimeter in the sand mold and metal mold for a given casting section size was higher for P_r_ than for P_c_.
(4) The graphite particles’ shape factors, involving the maximum and minimum size, were at a lower level for the metal mold solidification (1.2–1.3) compared to the sand mold casting (1.3–1.5), which expressed a higher compactness degree and had a lower dependence on the casting wall thickness.
(5) The convexity shape factor, which involves the difference between the real and convex perimeter of graphite particles and reaching 1.0 when P_r_ = P_c_, was higher for the metal mold (0.93–0.94 versus 0.91–0.92); there was a low dependence on the wall thickness variation and a good relationship between obtained data for these parameters.
(6) For the shape factor involving graphite particles area and the maximum size, the metal mold solidification had a higher graphite compactness degree, as this factor had higher values than in the sand mold casting: RSF = 0.68–0.7 versus 0.59–0.64. The wall thickness variation had a small effect on the sand mold, but practically no effect on the metal mold.
(7) The graphite nodularity level depended on the mold’s thermal properties: the solidification in the metal mold, with its higher cooling rate, led to a higher level of nodularity.
(8) The higher the considered graphite compactness degree level (shape factor increasing from 0.50 up to 0.80), the lower the resulting graphite nodularity for both the metal mold (39.1% versus 88.5%) and sand mold (32.3% versus 83.1%). It also increased the difference between the metal mold and sand mold regarding the average graphite nodularity in favor of the metal mold.

## Figures and Tables

**Table 1:** Chemical composition of tested cast iron * (mass%).

C	Si	Mn	P	S	Mg	Ce	La	Ca	Al	CE **
3.33	4.55	0.22	0.04	0.01	0.035	0.0004	0.0061	0.0038	0.0054	4.70

* Others elements (wt.%): 0.0067 Ti, 0.053 Cr, 0.10 Ni, 0.19 Cu, 0.072 Mo, 0.015 Sn, 0.019 As, 0.047 Sb, 0.0008 Bi, 0.003 Pb, 0.013 Co, 0.004 Nb, 0.008 V, 0.08 W and 0.019 N. ** Carbon equivalent calculated with Equation (1).

**Table 2:** Convex and real graphite particles perimeter in wedge castings solidified in green sand and metal molds.

Mold Type	Green Sand Mold					Metal Mold				
Casting Section size (mm)	6	9	12	15	Average	6	9	12	15	Average
Convex Perimeter (µm)	45.590	51.416	53.849	52.359	50.804	32.573	36.632	37.121	37.927	36.063
Real Perimeter (µm)	47.468	53.821	55.793	55.719	53.200	35.472	35.083	38.116	39.927	37.150
Pr/Pc Ratio	1.041	1.047	1.036	1.064	1.047	1.089	1.044	1.027	1.029	1.047
ΔP_1_ (Pr–Pc) (µm)	1.878	2.405	1.945	3.359	2.397	2.899	1.549	0.995	1.098	1.635

**Table 3:** Graphite shape factors.

Mold Type	Green Sand Mold					Metal Mold				
Casting Section Size (mm)	6	9	12	15	Average	6	9	12	15	Average
Aspect Ratio	1.394	1.355	1.336	1.359	1.361	1.240	1.265	1.295	1.248	1.262
Elongation	1.477	1.424	1.399	1.428	1.432	1.267	1.230	1.295	1.271	1.266
Convexity	0.911	0.916	0.922	0.911	0.915	0.933	0.934	0.935	0.937	0.935
Roundness Shape Factor	0.651	0.672	0.684	0.662	0.667	0.712	0.710	0.711	0.715	0.712

**Table 4:** Graphite nodularity (NG_2_) at imposed minimum values for graphite roundness shape factor in wedge castings for 4.5% Si uninoculated ductile iron that was solidified in a sand mold and a metal mold.

Mold	Wall Thickness, mm	Graphite Nodularity, NG_2_ (RSF), %			
		RSF, Minimum			
		0.50	0.60	0.65	0.80
Sand Mold	6	80.5	69.6	61.0	25.6
	9	82.9	70.0	65.3	32.1
	12	86.3	78.4	72.6	39.9
	15	82.8	69.1	63.2	31.7
	Average	83.1	71.8	65.5	32.3
Metal Mold	6	83.0	73.4	67.3	34.1
	9	87.1	77.8	72.6	37.8
	12	90.8	82.6	77.5	44.8
	15	93.0	84.4	77.3	39.6
	Average	88.5	80.0	73.7	39.1
ΔNG (MM-SM)		5.4	8.2	8.2	6.8

## Data Availability

Not applicable.

## Abbreviations

PMT	Photomultiplier tubes
CCD	Spectroscopic charge-coupled device
DI	Ductile iron
CE	Carbon equivalent
P_c_	Convex perimeter
Pr	Real perimeter
A_G_	Graphite area
D_Min/Mean/Max_	Minimum/mean/maximum diameter
F_Min/Mean/Max_	Minimum/mean/maximum Ferret
AR	Aspect ratio
E	Elongation
Cv	Convexity
WT	Wall thickness
SM	Sand mold
MM	Metal mold
RSF	Roundness shape factor
NG_1_	Graphite nodularity calculated with Equation (3)
NG_2_	Graphite nodularity calculated with Equation (4)
A_tot_	Area of all graphite particles
A_particle(RSF)_	Area of graphite particle at a minimum imposed RSF
